# Phylogenetics and species delimitations of the operculated land snail *Cyclophorus volvulus* (Gastropoda: Cyclophoridae) reveal cryptic diversity and new species in Thailand

**DOI:** 10.1038/s41598-019-43382-5

**Published:** 2019-05-07

**Authors:** Nattawadee Nantarat, Chirasak Sutcharit, Piyoros Tongkerd, Christopher M. Wade, Fred Naggs, Somsak Panha

**Affiliations:** 1 Animal Systematics Research Unit, Department of Biology, Faculty of Science, Chulalongkorn University, Bangkok, 10330 Thailand; 2 Environmental Science Research Center (ESRC), Chiang Mai University, Chiang Mai, 50200 Thailand; 3 Department of Biology, Faculty of Science, Chiang Mai University, Chiang Mai, 50200 Thailand; 4 School of Life Sciences, University of Nottingham, University Park, Nottingham, NG7 2RD UK; 5 Life Sciences Department, The Natural History Museum, London, SW7 5BD UK

**Keywords:** Molecular evolution, Phylogenetics, Bayesian inference, Zoology

## Abstract

Recent conceptual and practical advances in phylogenetic species delimitation have enabled progressively robust biodiversity studies. Delimiting species in widespread taxa is an intriguing problem; the edible operculated land snail *Cyclophorus volvulus* (Müller, 1774) is a good example since it shows a high degree of shell and color variation along with a widespread distribution throughout Thailand. Taxonomic boundaries for *C*. *volvulus* were examined and clarified using a combined morphological and phylogenetic approach, the latter of which was based on both nuclear and mitochondrial gene sequences. Moreover, three species delimitation analyses were applied: Poisson tree processes (PTP), automatic barcode gap discovery (ABGD), and generalized mixed Yule-coalescent (GMYC). All phylogenetic trees revealed that *C*. *volvulus* was polyphyletic and comprised of three clades that coincided with their geographic distribution. The three species delimitation analyses concurred with the phylogenies and formed at least three groups. According to the results, *C*. *volvulus* s.l., as currently recognized, consists of three distinct species in Thailand: *C*. *volvulus* s.s., *C*. *occultus* sp. nov., and *C*. *borealis* sp. nov., which are described herein. Moreover, several of these highly distinct *C*. *volvulus* evolutionarily significant units (ESU) are likely to require urgent conservation attention.

## Introduction

*Cyclophorus* Monfort, 1810 is an operculated land snail genus whose members are widely distributed in Southeast Asia^1–3^. Traditionally, *Cyclophorus* snails have been classified based only on their shell morphological characters^1–4^. However, shell characteristics can be extremely variable, a phenomenon caused by phenotypic plasticity^5–8^, especially in widely dispersed species^9^. *Cyclophorus* are among the most confused in terms of taxonomy. They are one of the largest and most diverse groups of Cyclophoridae, with more than 100 nominal species^1–4,6,7^. However, some *Cyclophorus* species show high morphological variation, which can make their identification very difficult.

*Cyclophorus volvulus* (Müller, 1774) is one *Cyclophorus* species that shows high morphological variability. It is widely distributed in limestone habitats in Asia, including India, Thailand, Malaysia, China, and Vietnam^1,3,6,10^.*C*. *volvulus* is common in Thailand and resides in almost all parts of the country^6,11,12^. For over 4,000 years, this snail has been considered edible by local Thai, Laotian, and Vietnamese people^13^. However, in the past ten years, the number of *Cyclophorus* species, including *C*. *volvulus*, appear to be declining^14^, particularly in Eastern and Central Thailand. This phenomenon could be caused by overharvesting, climate change, and/or habitat destruction, among others. The high degree of intra-specific variation in shell morphology and geographically disjunct populations may confound taxonomy, either by lumping different species or by oversplitting species on the basis of geographic population differentiation^6^. Thus, the taxonomic status of this snail remains muddled, and additional methods are required to help resolve the taxonomic status of *C*. *volvulus*.

Molecular phylogenetic analyses and species delimitation approaches are powerful tools for resolving taxonomy, including cryptic species. These techniques have aided in understanding the evolutionary history of several land snail taxa^6,7,9,15,16^. The molecular techniques are useful to identify valuable morphological characteristics or evaluate features that are inappropriate for taxonomy^17^. Thus, this study aimed to determine the validity of species boundaries in *C*. *volvulus* within Thailand by combining molecular phylogeny, species delimitation, and morphological approaches. The recognition of cryptic species is necessary to evaluate the true diversity and inform conservation management of the regional status of this snail in limestone habitats.

## Results

### Molecular and phylogenetic analyses

The 16S ribosomal RNA (rRNA) (462 bp), cytochrome oxidase I (COI) (660 bp), 28S rRNA (585 bp), and concatenated dataset of all three DNA regions (1,707 bp) were aligned for the 29 individual *C*. *volvulus* samples, which represented 15 populations from Thailand, along with *Cyclotus* spp. and *Leptopoma vitreum* as outgroups. The concatenated dataset had a 44.55% GC content. Heterogeneity in base composition between sequences is known to affect phylogenetic inference, so the variations in base pair composition among sequences for the concatenated datasets were tested using χ^2^ analysis (χ^2^ = 29.54, degrees of freedom = 120, P = 1.000). The dataset included 609 variable sites and 443 parsimony-informative sites. The partition homogeneity test, performed using PAUP 4.0b10 with 100 replicates, showed no significant incongruence between the gene fragments (P = 0.097), while the K2P-distance between the taxa (without outgroups) ranged from 0.000 to 0.120 (inter- and intra-specific K2P-distances were 0.079 and 0.012, respectively).

**Figure 1:**
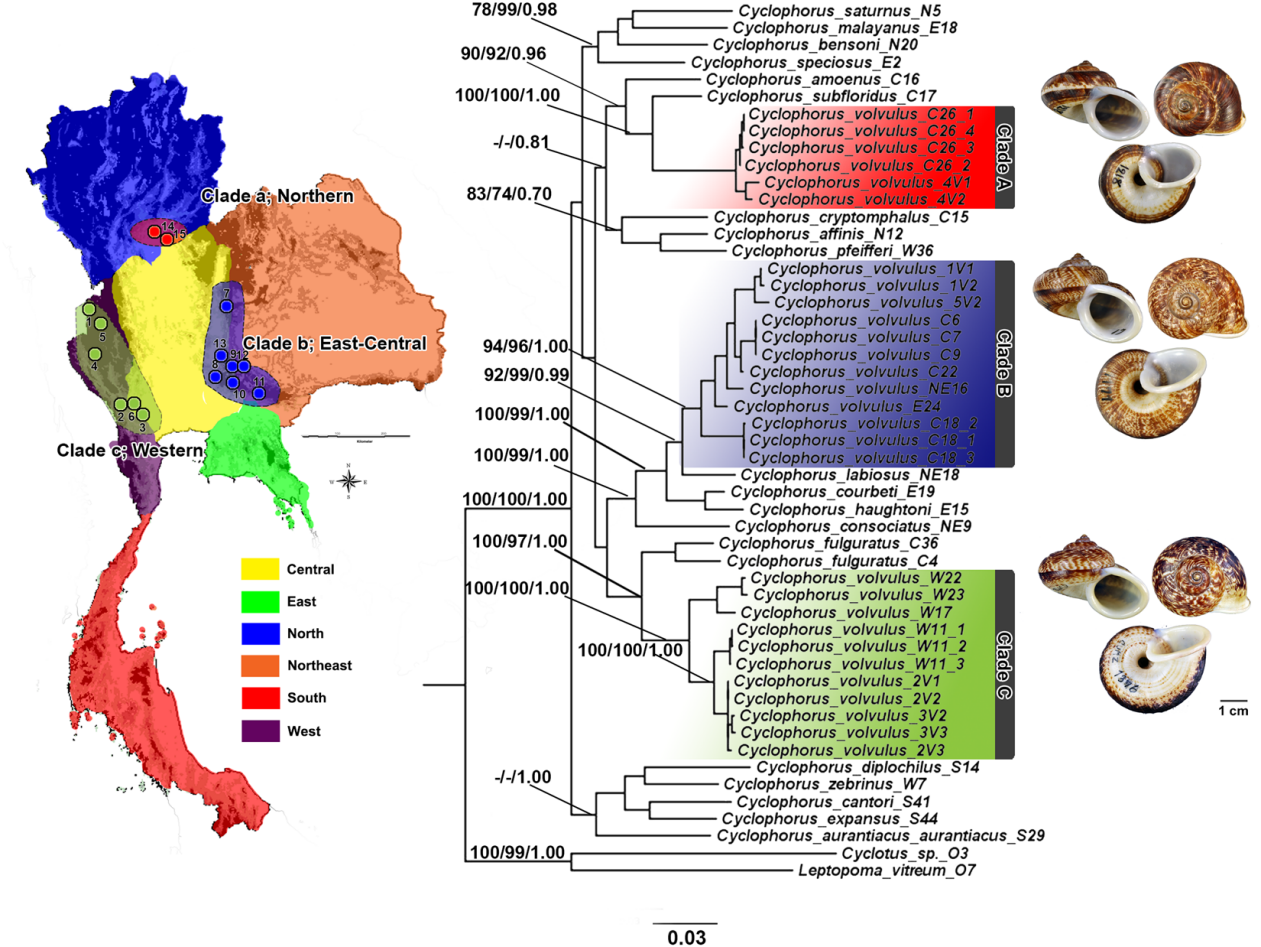
Location of *C*. *volvulus* complex sampling sites in Thailand and a BI tree. A phylogenetic tree of the *C*. *volvulus* complex and related species was constructed using 1707 nucleotide sites of the concatenated 16S, COI and 28S genes. Bootstrap support values (when >70%) and PP (when >0.70) for each node are shown on the tree (based on the NJ/ML/BI methods).

The phylogenetic relationships of *C*. *volvulus* samples are shown in Fig. 1. There was significant statistical support for a phylogenetic tree where certain clades coincided with certain morphological groups. However,*C*. *volvulus* was polyphyletic; it was divided into three well-supported main clades that represented three geographical regions, i.e., Northern (clade A), East-Central (clade B), and Western (clade C) Thailand. The sister taxon for clade A was *C*. *subfloridus*, while the sister taxa for clade B and C were *C*. *labiosus* and *C*. *fulguratus*, respectively. Clade A was strongly supported by 100% neighbor-joining (NJ) and maximum-likelihood (ML) bootstrap replicates and 1.00 posterior probability (PP) of Bayesian inference (BI). Clade B was supported by 94% NJ and 96% ML bootstrap replicates and 1.00 PP of BI, and clade C by 100% of NJ and ML bootstrap replicates and 1.00 PP of BI (Fig. 1). The K2P-distances among clades A, B, and C ranged from 0.104–0.113 for 16S rRNA, 0.146–0.152 for COI, and 0.008–0.019 for 28S rRNA (Tables 1 and 2).

**Table 1:** Range of genetic divergence (K2P-distance) of the three *C*.

	Clade A	Clade B	Clade C
Clade A: Northern	—	0.151	0.152
Clade B: East-Central	0.113	—	0.146
Clade C: Western	0.104	0.087	—

*Volvulus* clades (A–C) and related species based on the mitochondrial 16S rRNA gene (below diagonal) and COI (above diagonal) sequences.

**Table 2:** Range of genetic divergence (K2P-distance) of the three *C*.

	Clade A	Clade B	Clade C
Clade A: Northern	—		
Clade B: East-Central	0.008	—	
Clade C: Western	0.019	0.016	—

*Volvulus* clades (A–C) and related species based on the nuclear 28S rRNA gene (below diagonal) sequence.

### Species delimitation

**Figure 2:**
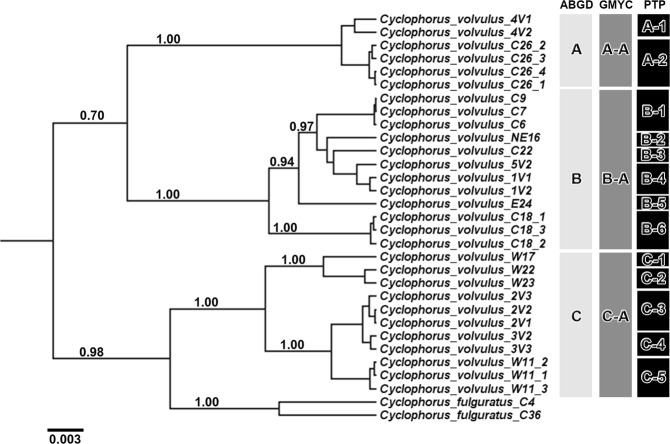
Species delimitation analyses of the *C*. *volvulus* complex based on the concatenated 16S rRNA, COI and 28S rRNA genes. Results of species delimitation analyses using ABGD^41^, GMYC^42^ and PTP^16^, respectively. PSHs are labeled with letters and numbers; The PP support values for each node are shown on this BI phylogenetic tree when greater than 0.70.

The three Thai *C*. *volvulus* groups (clades A, B, and C; Fig. 1) were supported by all three species delimitation methods (GMYC, ABGD, and PTP). Moreover, the three methods were congruent and proposed the three *C*. *volvulus* clades as candidate species, hereafter denoted as Group-A, Group-B, and Group-C (Fig. 2). However, the PTP method went further by splitting the three clades into more restricted candidate species.

### Geometric morphometrics of shell morphology

**Figure 3:**
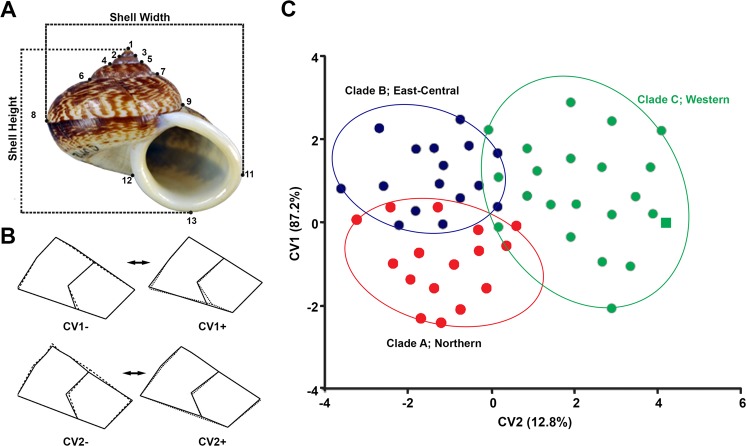
Geometric-morphometric study of shell shape variation in *C*. *volvulus* (type specimen; square symbol). (**A**) The measurements and landmarks (13 black spots). (**B**) Wireframe pictures of *C*. *volvulus* shell showing the shape deformations (solid line) from the consensus configuration (dotted line) to each extreme negative and positive CVs. (**C**) Plots of individual scores for the canonical variates (CVs) that were derived from the canonical variate analysis (CVA).

Clades A–C (Fig. 1) were provided as an *a priori* group for each canonical variate analysis (CVA), which supplied a graphical display of the shape differences between them from the relationship between the two obtained canonical variate (CV) variables (Fig. 3). The shape variability explained 66.64% of the variance in the first canonical axis (CV1), with an eigenvalue of 0.7039, while shell shape variability explained 12.78% of the variance in the second axis (CV2), with an eigenvalue of 0.8358. Overall, the shell shapes of the three clades showed some overlap. However, there were also significant differences among clades based on permutation tests for Mahalanobis and Procrustes distances (P < 0.0001 to 0.0018 for *C*. *volvulus*). Accurate shell classification for each clade, along with cross-validation of discriminant function analysis, was 78% for clade A, 67% for clade C, and 63% for clade B (Fig. 1), with a 69% overall rate of reliability for identifying each *C*. *volvulus* individual. The overall significance tests were based on pooled variances (i.e., the average variance across all groups).

A wireframe modification (Fig. 3) defined the shape change from the consensus configuration along the two CVs. For CV1, individuals located in the positive portion of the axis had a wider shell compared to those in the negative portion (represented by shifts in landmarks 6 and 8 for *C*. *volvulus*). CV1 showed differences in aperture, where individuals in the positive portion of the axis had a narrower shell aperture compared to the negative portion. The CV2 of the *C*. *volvulus* complex showed differences in shell shape, prominently defined by landmarks 7 and 9, which represent the side of the last whorl twist, compared to the other landmarks as the score decreased.

**Figure 4:**
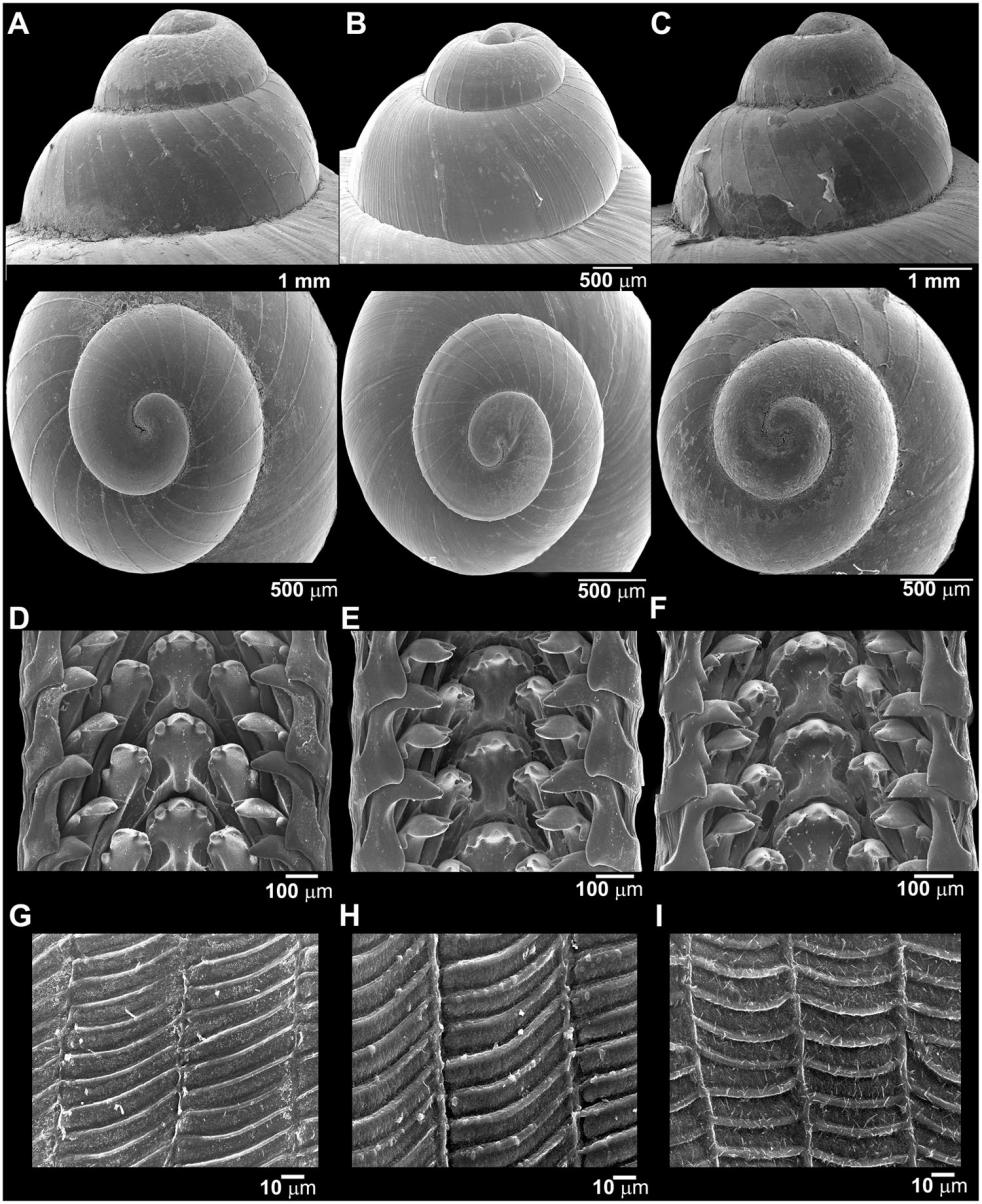
Representative SEM images of the side and top views of the protoconch (**A**–**C**), radula morphology (**D**–**F**) and surface sculpture of jaw (**G**–**I**) for *C*. *volvulus* specimen (**A**,**D**,**G**) CUMZ 1376 (Clade C), (**B**,**E**,**H**) CUMZ 1171 (Clade B) and (**C**,**F**,**I**) CUMZ 1281 (Clade A).

In the present study, most of the examined *C*. *volvulus* samples showed a high degree of similarity in shell size and protoconch and jaw patterns (Fig. 4). However, we found slightly different characteristics among clades A–C, such as shell and umbilicus color patterns. However, the possible type specimen was placed in clade C; its shell was most similar to *C*. *volvulus*.

## Discussion

In this study, we confirmed the existence of three highly divergent *C*. *volvulus* s.l. lineages in Thailand; these genetically differentiated and geographically isolated populations were confirmed using multi-loci species delimitation. These lineages (Clades A, B and C in Northern, East-Central, and Western Thailand, respectively; Fig. 1) were first recognized in a previous study^6^, and further verified here with the analysis of new samples as highly divergent populations (Figs 1 and 2). Therefore, the three gene loci that we analyzed were generally consistent with the previous study on the relationship of the genus *Cyclophorus*^6^. The individual gene trees showed some differences in topology and support for each lineage. However, individual gene trees are often different from the species tree due to retention and incomplete sorting of ancestral polymorphisms, especially in the case of recently diverged species or populations^6,18–20^. All three species delimitation analyses revealed the three *C*. *volvulus* lineages identified in the phylogenetic trees (Fig. 1) and clearly reflected three-to-thirteen distinct evolutionary significant units (ESUs; Fig. 2). PTP species delimitation analysis revealed additional candidate species within *C*. *volvulus* relative to the ABGD and GMYC methods (number of candidate species: PTP = 13, ABGD = 3, and GMYC = 3). Intra-specific K2P divergences for mitochondrial DNA in the three main candidate species ranged from 0.011–0.041, while the other *Cyclophorus* species ranged from 0.000–0.021. These species delimitation analyses were responsible for the high average intra-specific K2P genetic divergence observed for all candidate species. This relatively high intra-specific genetic divergence suggests that there are at least three separate species (clades A–C). Additionally, the results highlight differences in evolutionary patterns for the three nucleotide regions during inter-specific divergence and following demographic changes in candidate species. Notably, similar genetic distances were inferred to be indicative of distinct species in studies of other land snails^7,21^.

Generally, cryptic diversity is common among land snails that are widely distributed and/or fragmented in distribution^12^. As time passes reduced gene flow and/or a complete lack of genetic exchange between populations would promote new species due to allopatric processes. Indeed, morphology comparison among the three clades with their sister taxa underscored this supposition. Specifically, clade A was similar to *C*. *subfloridus* in aperture shape but different in shell shape, size, and color pattern, clade B was similar to *C*. *labiosus* in color pattern but different in aperture shape, and clade C was similar to *C*. *fulguratus* in shell size but different in color pattern.*C*. *volvulus* clades A–C (Fig. 1) and their sister taxa may have recently undergone allopatric fragmentation within the large geographic distribution between the different localities^7,22^. Alternatively, they may be under similar selective pressure or niches that cause them to undergo convergent evolution^23^. Intriguingly, our PTP analysis provided a different number of candidate species compared to ABGD and GMYC methods. This discrepancy may demonstrate that neutral genetic variation in the snail is influenced by the environment. Compared to the three consistent candidate species identified with all delimitation methods, the remaining ten PTP-derived candidate species exhibited relatively little difference in shell morphological characteristics (e.g., size, shape, and color pattern). We conclude that the intra-specific genetic divergence for these additional PTP-identified species is too low to currently consider them as distinct species^7,21^, but the relatively high genetic divergence indicates they may split into different species in the future.

Although the external *C*. *volvulus* s.l. morphology among the three clades was highly similar, there were slight differences. Shell shape geometric morphometrics revealed significant divergence among these groups as well as considerable overlap in the shell shape, results that support their cryptic morphology. The possible type specimen, considered most related to *C*. *volvulus* in shell shape, was placed in clade C. In addition to the high degree of external morphology similarity, the genital systems of these groups also could not be used for classification and identification^6,11^.

In this study, we provided a robust provision for the previously reported relationships and conservation management of *Cyclophorus*^6^. We also identified priorities for ongoing and future investigations. Further, an extensive taxonomic revision of this genus would be valuable. The evidence presented in this study strongly suggests that the three geographic populations of the *C*. *volvulus* complex in Thailand should be recognized as three distinct species: *C*. *volvulus* s.s. (clade C) and the undescribed *C*. *occultus* sp. nov. (clade B), and *C*. *borealis* sp. nov. (clade A).

## Systematics

Family *Cyclophoridae* Gray, 1847

Genus *Cyclophorus* Montfort, 1810

*Cyclophorus volvulus* (Müller, 1774)

Figure 1 (clade C), 4 (A, D, G) and 5 (A, B)

*Helix volvulus* Müller, 1774: 82. Type locality: unknown.

*Cyclophorus volvulus* — Kobelt, 1911: 143, 144^1^. Nantarat *et al*., 2014: 99–111, Figs 3 and 4B (clade 1v)^6^.

### Material examined

**Figure 5:**
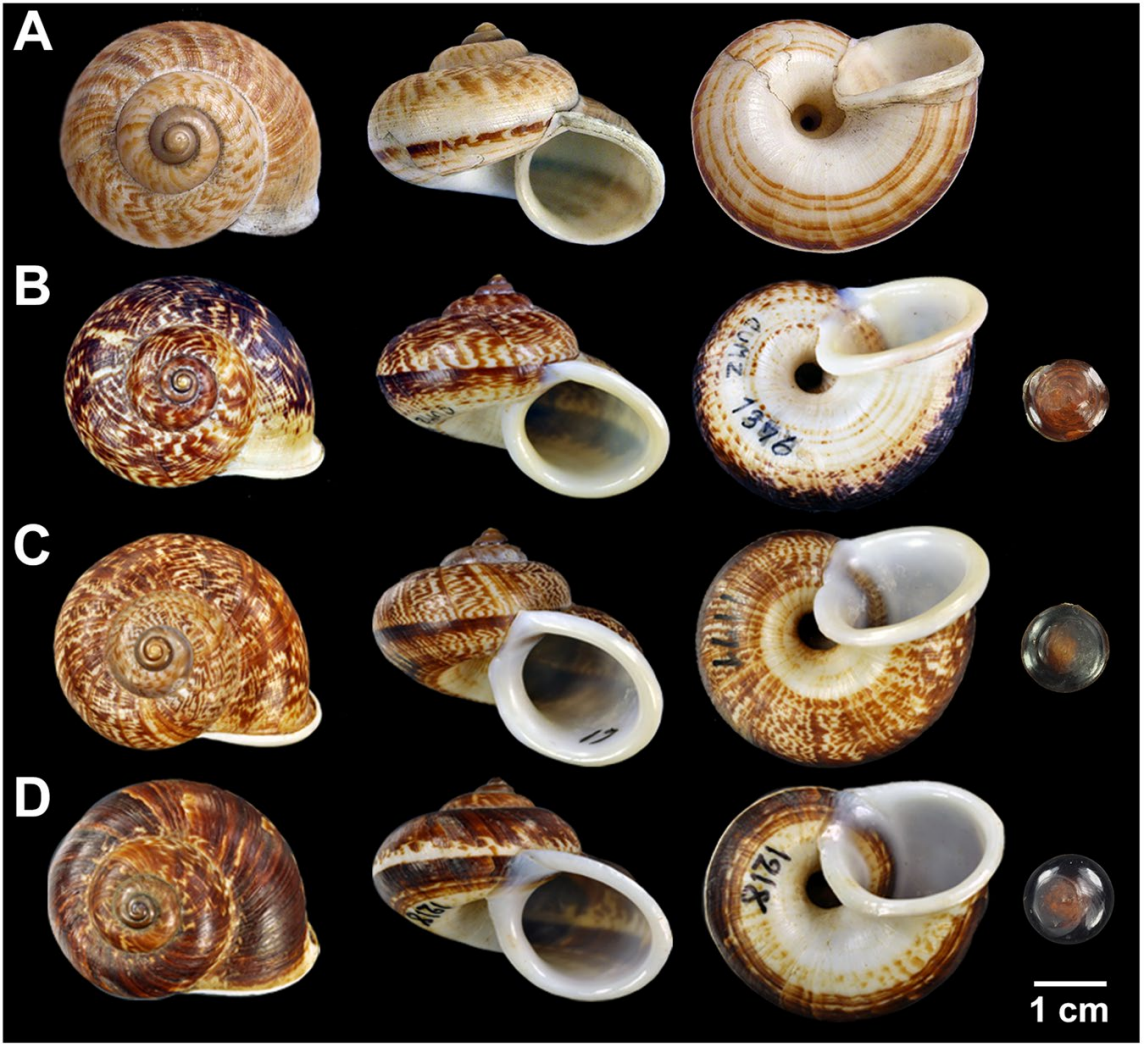
Shell of *Cyclophorus* species. (**A**,**B**) *Cyclophorus volvulus* s.s. (**A**) Possible Syntype ZMUC ex. Spengler coll., and (**B**) specimen CUMZ 1376/1 (Clade C in Fig. 1). (**C**) *C*. *occultus* sp. nov. (clade B in Fig. 1) holotype CUMZ 1771, and (**D**) *C*. *borealis* sp. nov. (clade A in Fig. 1) holotype CUMZ 1218.

Possible syntypes ZMUC ex. Spengler coll. (1 shell; Fig. 5A). Lan-Sang, Tak: CUMZ 1376 (3 shells) [N 16°46′57″ E 99°01′08″], CUMZ 1669 (18 shells) [N 16°46′57″ E 99°01′08″], Wang Chao, Tak Province: CUMZ 1749 (2 shells)[N 16°30′16″ E 99°09′40″], Doi Hau Mod, Tak Province: CUMZ 1747 (20 shells) [N 15°57′36.5″ E 98°51′22.1″], Erawan Waterfall, Kanchanaburi Province: CUMZ 1830 (2 shells) [N 14°22′30.9″ E 99°08′39.4″], Khao Rong, Phetchaburi: CUMZ 1713 (4 shells) [N 13°01′14″ E 99°54′53″], Aow Noi, Prachuapkhiri Khan: CUMZ 1806 (11 shells) [N 11°51′39.2″ E 99°49′18″].

### Measurements

Shell height: ranges 23–27 ± 0.03 mm Shell width: ranges 29–37 ± 0.03 mm.

### Description

Shell medium, thick, solid with low conical shape, smooth or with variously developed spiral ribs. Periostracum thin, with white umbilicus and narrow white spiral band on the last whorl. Aperture round or oblique; lip whitish with expanded and reflected. Umbilicus rather wide and deep. Operculum thin corneous, round, dark brown with multispiral.

### Radula and jaw

Taenioglossan radula, each row contains 7 teeth with formula 2-1-1-1-2. Central tooth symmetrical semicircular shape composed of 5 denticles; central cusp well developed, largest and pointed tip; inner lateral cusps small, triangular shape with pointed cusp; outer lateral cusps very small with dull cusps. Lateral and inner marginal teeth tricuspid consisted 3 cusps; central cusp large, convex shape, and flanked with smaller and pointed head of one inner and one outer lateral cusps. At the edge of ribbon, outer marginal teeth bicuspid consisted 2 cusps; inner cusp small with pointed head; outer cusp very large triangular shape with pointed (Fig. 4D).

Jaw consists of two symmetrical parts, each part thin and rhomboid shape. Sculpture with very thin longitudinal parallel ridges, connected to each other with strong parallel slanting transverse ridges, which made sculpture of jaw like underside of leaf blade (Fig. 4G).

### Distribution

The ranges of this species are demarcated to Southeast Asia including Southern China, Hong Kong and India.

*Cyclophorus occultus* Nantarat and Panha, sp. nov.

urn:lsid:zoobank.org:act:63358F60-1801-4A13-B75C-AA1857370309

Figures 1 (clade B), 4(B,E,H) and 5C

*Cyclophorus volvulus* — Nantarat *et al*., 2014: 99–111, Figs 3 and 4B (clade 2v)^6^.

### Type materials

Holotype CUMZ 1171 (Fig. 5C; height 25 mm, width 32 mm, 5 whorls). Paratypes CUMZ 1762 (17 shells) and NHMUK (2 shells).

### Type locality

Sao Wa Luk camp, Nakhonratchasima Province (N 14°35′45″ E 101°23′31″).

### Etymology

The specific epithet ‘*occultus*’ is from the Latin word meaning “hidden or, concealed” with reference to the cryptic species. Description of this new species is here attributed to the first and the last author, Nantarat and Panha, respectively.

### Other material examined

Wang Kan Luang Waterfall, Lopburi Province: CUMZ 1618 (12 shells) [N 15°6′48″ E 101°6′48″], Tum Sun Ti-Suk Temple, Lopburi Province: CUMZ 1784 (3 shells) [N 15°12′10″ E 100°39′50″], Dao Khao Kaeo Cave, Saraburi Province: CUMZ 1716 (3 shells) [N 14°53′05″ E 101°20′49″], Ban Ta Sao, Saraburi Province: CUMZ 1617 (3 shells) [N 14°42′16.8″ E 101°4′43.4″], Phu-Kae Botanic Garden, Saraburi Province: CUMZ 1629 (17 shells) [N 14°40′24″ E 100°53′22″], Khao Chakan, Sra Kaeo Province: CUMZ 1756 (15 shells) [N 13°39′28.9″ E 102°05′18.6″].

### Measurements

Shell height: ranges 23–25 ± 0.02 mm. Shell width, ranges 30–34 ± 0.05 mm.

### Description

Shell medium, thick, solid with low conical shape, apex acute; whorls 5 to 6, convex; suture deep and wide. Periostracum exhibits color variation from dark to pale brown. Protoconch with thin narrowly discernible transverse ridges (Fig. 4B). Last whorl rounded, enlarge and with whitish colour. Shell colour usually light to dark brownish colour, sometime dark zigzag streak present; on periphery with narrow dark spiral band; lower periphery with wide striated stripe surrounded umbilicus. Aperture circular, slightly oblique; lip little expanded and reflected with white colour. Umbilicus widely open and deep. Operculum corneous, multi-spiral and little concaved center (Fig. 5C).

### Radula and jaw

Central tooth big and semicircular with 5 denticles, large at center and smaller arrangement of the other. Lateral teeth bicuspid, endocone small with sharp cusp, ectocone large. Marginal teeth bicuspid, have different arrangement into two layers of inner marginal teeth locates the same level of central tooth, the large sharp inflated ectocone, the outer marginal teeth locates at the edge of the ribbon with a little lower position from lateral teeth, with almost the same shape as inner marginal teeth.

General structure of jaw similar to that of *C*. *volvulus* s.s. (Fig. 4H) The difference characters are sculptured with thicken and strong longitudinal parallel ridges and connected to each other with very strong parallel slanting transverse ridges (Fig. 5C).

### Distribution

This new species is known from several localities in the northeastern Thailand. The application of the taxon name in previously publications by Nantarat *et al*.^6^ as the *C*. *volvulus* s.l. are here reconsidered as the new species.

### Remark

This new species can be distinguished from *C*. *volvulus* s.s. and *C*. *borealis* sp. nov. by having uniform brown to brownish shell colour, with light brown zigzag streaks. Aperture lip is a little bit more oblique than *C*. *volvulus* s.s. Shell pattern around umbilicus with wide spiral band and light brown zigzag streaks almost umbilicus area. This new species also supported by phylogenetic tree (Fig. 1) and geometric morphometrics (Fig. 3).

*Cyclophorus borealis* Nantarat and Panha, sp. nov.

urn:lsid:zoobank.org:act:62369102-DE9A-4323-B420-3E54AD961922

Figures 1 (clade A), 4(C,F,I) and 5D

*Cyclophorus volvulus* — Nantarat *et al*., 2014: 99–111, Figs 3 and 4B (clade 3v)^6^.

### Type materials

Holotype CUMZ 1218 (Fig. 5D; height 25 mm, width 31 mm, 5 whorls). Paratypes CUMZ 1673 (22 shells) and NHMUK (2 shells).

### Type locality

Tum Ra Kang, Sukhothai Province (N 17°09′54″ E 99°33′35″).

### Etymology

The specific epithet is from the Latin word ‘*boreas*’ meaning “northern”. It refers to the new species that strictly appears in northern Thailand. Description of this new species is here attributed to the first and the last author, Nantarat and Panha, respectively.

### Other material examined

Tum Ra Kang, Sukhothai Province: CUMZ 1673 (24 shells) [N 17°09′54″ E 99°33′35″], Pa Ma-Muang Temple, Phitsanulok Province: CUMZ 1770 (15 shells) [N 16°34′1.8″ E 100°40′30.4″].

### Measurements

Shell height: 24–26 ± 0.04 mm. Shell width, ranges 29–37 ± 0.07 mm.

### Description

Shell medium, funnel-shaped, umbilicate, solid, smooth surface, brownish, apex acute; whorls 5, convex; suture deep and wide. Periostracum thick corneous with brownish colour. Protoconch with thin closely discernible transverse ridges (Fig. 4C). Remain whorls with only thin irregular growth lines. Last whorl rounded, expand, with white colour background. Shell colour brownish, with dark zigzag streak present; on periphery with narrow dark brown spiral band; on lower periphery with narrow striated stripe surrounded umbilicus. Aperture circular, slightly oblique; lip little expanded with white colour. Umbilicus widely open and deep. Operculum corneous, multi-spiral and little concaved center (Fig. 5D).

### Radula and jaw

Central tooth semicircular 5 denticles with large center and smaller arrangement of the other. Lateral teeth bicuspid, endocone small with sharp cusp, ectocone large. Marginal teeth bicuspid, have different arrangement into two layers of inner marginal teeth locates the same level of central tooth, the large sharp inflated ectocone, the outer marginal teeth locates at the edge of the ribbon with a little lower position from lateral teeth, with almost the same shape as inner marginal teeth (Fig. 5F).

General structure of jaw similar to that of *C*. *volvulus* s.s. The difference characters are sculptured with strong longitudinal parallel ridges, connected to each other with solid parallel curving transverse ridges (Fig. 5I).

### Distribution

This new species is known from the northern Thailand. The application of the taxon name in previously publications by Nantarat *et al*.^6^.

### Remark

This new species can be distinguished from *C*. *volvulus* s.s. and *C*. *occultus* sp. nov. by having uniform brown to dark brownish shell colour, with dark brown zigzag streaks. The last whorl is relatively larger than *C*. *volvulus* s.s. and *C*. *occultus* sp. nov. Shell height is relatively more than the other two species. Shell pattern around umbilicus with solid band outside and light brown spiral line with light brown zigzag streaks about ¾ of umbilicus. This new species also supported by phylogenetic tree (Fig. 1) and geometric morphometrics (Fig. 3).

## Conclusion

Morphologically cryptic species among land snails are an important problem for taxonomists, ecologists, and biogeographers. Study of these species provides valuable information for communicating the unique properties of separate evolutionary lineages and conservation management. The presented phylogenetic interpretation of *C*. *volvulus* s.l., based on mtDNA (16S rRNA and COI) and nuDNA (28S rRNA) sequence data, was conducted using multiple approaches, including molecular phylogeny, species delimitation, geometric morphometrics, and morphological data. All methods supported the presence of at least three geographically distinct candidate species in *C*. *volvulus* s.l. over broad regions (clades A, B, and C from Northern, East-Central, and Western Thailand, respectively). We clarified the species boundaries of *C*. *volvulus* and the two newly described species. We unveiled cryptic diversity in a biodiversity hotspot: the identification of these (at least) three cryptic lineages suggests additional species diversity. The new species may comprise the portion of the potential niche that is actually occupied, given that historical and biotic factors pose further restriction on new species’ distribution^24^. Differences among new species’ niches could reflect their evolution, an actual change in the potential niche, and/or a mechanism that results in isolation and speciation. However, these differences could also reflect morphological plasticity, where some PTP candidate species possess the same potential niche despite ostensibly expressing a different niche. This study also provides useful information to evaluate ESUs for conservation, especially in some parts of Northeast and East-Central Thailand where the snail populations have declined dramatically. Understanding the high level of lineage diversity in an operculated land snail in Thailand, is within an area designated as one of the biodiversity hotspots and a world conservation priority^25^.

## Methods

### Taxon sampling and morphology

*Cyclophorus volvulus* s.l. was collected from 15 populations (Tables 3 and 4). Species determination was based on the publications of Kobelt^1^, Reeve^3^, Müller^10^, and Benthem Jutting^26,27^. The shells were subsequently compared with the relevant type specimens and reference collections, including those at the Natural History Museum of Denmark (Zoological Museum), University of Copenhagen, Denmark (ZMUC), The Natural History Museum, London (NHMUK), and Chulalongkorn University, Museum of Zoology, Bangkok, Thailand (CUMZ). Living specimens were preserved. The foot tissues were fixed and preserved in 95% (v/v) ethanol, while the remaining parts of each specimen were preserved in 70% (v/v) ethanol for morphological study. In total, 51 specimens of *C*. *volvulus* and related species were examined. Adults shell were measured with Vernier calipers to the nearest 0.1 mm and photographed with a digital camera.

**Table 3:** Localities of *Cyclophorus volvulus* samples and GenBank accession no. of the COI and 16S rRNA and 28S rRNA sequences.

Map No.	Species/Locality	Number of specimens		
		16S rRNA	COI	28S rRNA
	***C***. ***volvulus*** **(clade C; Western)**			
1	Lan-Sang, Tak	MH644649	MH644632	MH644666
2	Khao Rong, Phetchaburi	JX474711	JX474585	KF319149
3	Aow Noi, Prachuapkhiri Khan	JX474709-10	JX474583-4	KF319147-8
4	Erawan waterfall, Kanchanaburi	JX474712, MH644664-5	JX474586, MH644647-8	KF 319150, MH644681-2
5	Wang Chao, Tak	MH644650	MH644633	MH644667
6	Doi Hau Mod, Kanchanaburi	MH644658	MH644641	MH644675
	***C***. ***volvulus*** **(clade B**, **East-Central)**			
7	Wang Kan Luang waterfall, Lopburi	JX474657-9	JX474603-5	KF319167-9
8	Dao Khao Kaeo cave, Saraburi	JX474688	JX474606	KF319170
9	Sao Wa Luk camp, Nakhonratchasima	JX474690	JX474607	KF319171
10	Ban Ta Sao, Saraburi	JX474791, MH644662-3	JX474609, MH644645-6	KF319173, MH644679-80
11	Khao Chakun, Sa Kaeo	JX474689	JX474608	KF319172
12	Phu-Kae moutain, Saraburi	MH644651-2	MH644634-5	MH644668-9
13	Tum-Sun-Ti-Suk temple, Lopburi	MH644653-5	MH644636-8	MH644670-2
	***C***. ***volvulus*** **(clade A**, **Northern)**			
14	Tum Ra Kang, Sukhothai	JX474668, MH644659, MH644660-1	JX474602, MH644642-4	KF319166, MH644676-8
15	Pa-Ma-Muang chapel, Phitsanulok	MH644656-7	MH644639-40	MH644673-4

**Table 4:** Localities of *Cyclophorus* spp. and outgroup samples and GenBank accession no. of the COI and 16S rRNA and 28S rRNA sequences.

Species	GenBank accession no.		
	16S rRNA	COI	28S rRNA
*C*. *aurantiacus aurantiacus*	JX474723	JX474642	KF319206
*C*. *affinis*	JX474678	JX474587	KF319151
*C*. *amoenus*	JX474660	JX474595	KF319159
*C*. *bensoni*	JX474671	JX474572	KF319136
*C*. *cantori*	JX474717	JX474628	KF319192
*C*. *consociatus*	JX474703	JX474620	KF319184
*C*. *courbeti*	JX474693	JX474611	KF319175
*C*. *cryptomphalus*	JX474665	JX474594	KF319158
*C*. *diplochilus*	JX474715	JX474624	KF319188
*C*. *expansus*	JX474719	JX474630	KF319194
*C*. *fulguratus*	JX474705, JX474708	JX474579, JX474582	KF319143, KF319146
*C*. *haughtoni*	JX474696	JX474614	KF319178
*C*. *labiosus*	JX474692	JX474610	KF319174
*C*. *malayanus*	JX474653	JX474568	KF319132
*C*. *pfeifferi*	JX474683	JX474591	KF319155
*C*. *saturnus*	JX474676	JX474562	KF319126
*C*. *subfloridus*	JX474666	JX474600	KF319164
*C*. *zebrinus*	JX474721	JX474632	KF319196
*Cyclotus* sp.	JX474739	JX474649	KF319213
*Leptopoma vitreum*	JX474741	JX474650	KF319214

Details of the shell surface, protoconch, jaw and radula morphology (form and formula of radula teeth) were observed using scanning electron microscopy (SEM; JSM-5410 LV) at the Scientific and Technological Research Equipment Centre (STREC), Chulalongkorn University.

### DNA extraction, amplification and sequencing

Genomic DNA was extracted from the foot tissue using a DNAeasy Tissue Kit (QIAGEN Inc.). The mitochondrial 16S ribosomal RNA (16S rRNA) region and cytochrome c oxidase subunit I (COI) gene region were PCR amplified using the 16sar and 16sbr^28^ and the LCO1490 and HCO2198^29^ primer pairs, respectively. The nuclear 28S ribosomal RNA (28S rRNA) region was PCR amplified using primers 28SF4 and 28SR5^30^. The thermal cycling conditions for amplification of the 16S rRNA and 28S rRNA were 2 min at 94 °C, followed by 36 cycles of 30 s at 94 °C, 30 s at 50 °C and 90 s at 72 °C, and then a final 5 min at 72 °C. That for the amplification of COI was performed in the same condition except that the annealing stage was changed to 2 min at 42 °C and the extension time to 2 min. Products were resolved and visually checked by 1% (w/v) agarose gel electrophoresis with SYBR Safe staining and blue light transillumination. A QIAquick purification Kit (QIAGEN Inc.) was used to purify the PCR products before being sent for commercial automatic cycle-sequencing at Macrogen, Inc. (Seoul, South Korea).

### Phylogeny inference

The mitochondrial DNA (approximately 462 bp of 16S rRNA and 660 bp of COI) and nuclear DNA (approximately 585 bp of 28S rRNA) sequences were edited and aligned using MUSCLE version 3.6^31^, a sub-program within the MEGA 6.01 software^32^, and then improved manually. The unambiguous of 16S rRNA, COI (all codon positions) and 28S rRNA fragments were aligned across all samples. All base frequencies and molecular character statistics were calculated using MEGA 6.01^32,33^.

All sequences were checked for ambiguous nucleotide sites and saturation before being subjected to phylogenetic analysis. The saturation graphs were plotted using uncorrected pairwise distances for transition and transversion substitutions to envision saturation and identify the taxa responsible. In the cases of accuracy of the combined data suffered relative to the individual partitions, the concatenate datasets of mitochondrial (16S rRNA and COI) and nuclear DNA (28S rRNA) were checked using the partition homogeneity test in PAUP 4.0b10, using 100 replicates^34^. The alignment was partitioned by gene fragments. However, the analyses were run for each gene fragment and also for the concatenated dataset. jModeltest 2.1.7^35^ was used to calculate and select a GTR + G model that was then used in all analyses.

Phylogenetic trees (Fig. 1) were constructed using neighbor joining (NJ), maximum likelihood (ML) and Bayesian inference (BI). The parameters, including the rate matrix and gamma shape parameter (α) of the gamma distribution (based on 16 rate categories) were estimated. The alignment was partitioned by gene fragment. The NJ analysis was performed using PAUP* v4.0b10^34^, while the ML analysis was performed via the RAxML Web servers^36^. Bootstrap resampling^37^ with 1000 replicates was used to assess branch support. The BI analysis was performed using MrBayes version 3.2.5^38^, where the tree space was explored using four chains of a Markov chain Monte Carlo algorithm (MCMC) and the optimum substitution model found above. The Bayesian analysis was run for 10 million generations (heating parameter = 0.03), sampling every 100 generations and then discarded 25% of the trees with burn-in. Convergence was monitored by showing the average standard deviation of the split frequencies (between 2 runs) was below 0.1%^38^. Support for nodes was defined as posterior probabilities (PP).

### Species delimitation methods

Three species delimitation methods (Fig. 2) were performed^39,40^, since the support across multiple species delimitation approaches is generally superior to a single method^40^. Thus, the species boundaries of *C*. *volvulus* were investigated based on the (i) Poisson Tree Processes (PTP)^16^, (ii) Automatic Barcode Gap Discovery (ABGD)^41^ and (iii) Generalized Mixed Yule-Coalescent (GMYC)^42,43^ methods. While ABGD detects ‘barcode gaps’ in the distribution of pairwise genetic distances, GMYC and PTP use an input tree as a model to delineate the PSHs in speciation and coalescent processes^40^. The ABGD approach was run using the web server (http://www.abi.snv.jussieu.fr/public/abgd/abgdweb.html) and the default parameters. The PTP and GMYC^43^ methods were also performed on the concatenated 16S rRNA, COI and 28S rRNA genes.

The delimitated species tree was produced using the relaxed log-normal clock algorithm implemented in the BEAST v1.8.2 package^44^. The GTR + G model was applied to re-construct the tree for 1 × 10^7^ generations with sampling every 100 steps. The MCMC output was examined by consideration of traces in Tracer 1.6^45^ and analyzed with TreeAnnotator 1.7.4 using all trees. A PP limit of 0.5 with maximum clade credibility tree was set. The GMYC method was performed using both a single- and a multiple-threshold. The output tree was optimized using SPLITS v.1.0–19 package for R. The PTP method was implemented based on the output tree and run in Python using the Environment for Tree Exploration package^46^.

### Geometric morphometric analysis

Photographs of 108 shells, including the shell-only collections and the 42 individuals for which we assembled the DNA sequence data from, were randomly ordered in tpsUtil v.1.49^47^. The shell photos were then digitized in tpsDig v. 1.40^48^ to capture the bi-dimensional coordination of 13 landmarks (Fig. 3A). Landmark coordinates for all specimens were evaluated in the software package MORPHOJ v1.06d^49^. All landmark data were superimposed by Procrustes Fit, with the data transformed into a common coordinate system to eliminate other variations, such as isometric size variation and orientation, except for shape from the data^47^. The allometric components/and the other effects of allometric shape variation were removed by using MORPHOJ v1.06d^49^. All statistical analyses were performed using MorphoJ^49^. Shell shapes were plotted against the centroid size which used as proxy of shell size to perform. Multivariate regression was used to discover any correlation among the centroid size and shape variable (p < 0.001; 10,000 replicates of permutation test) and its statistical significance. This allowed us to identify the modes of change that account for the highest degree of variation and provided linear numerical values for further analysis. Canonical variance analysis (CVA) was used to test the shell shape variation with candidate species recognized by molecular analyses as an *a priori* group. Mahalanobis and Procrustes distances between pairwise comparisons were calculated for significant differences by the permutation test (10,000 iterations). The correct classification percentage of each paired species was evaluated using the leave-one-out cross-validation, which was the implement of discriminant function analysis.

### Nomenclatural acts

The electronic edition of this article fit with the requirements of the amended International Code of Zoological Nomenclature (ICZN), and hence the new names contained herein are available under that the Code rules. The published work and the nomenclatural acts it encloses have been registered in ZooBank (http://zoobank.org), the online registration system for the ICZN. The LSID for this publication is: urn:lsid:zoobank.org:pub:458492DC-E3F5-4293-AE27-FFCAB7287FC5. The electronic edition of this paper was published in a journal with an ISSN, and has been recorded and is available from PubMed Central.
